# USP16 Downregulation by Carboxyl-terminal Truncated HBx Promotes the Growth of Hepatocellular Carcinoma Cells

**DOI:** 10.1038/srep33039

**Published:** 2016-09-16

**Authors:** Yu Qian, Boshi Wang, Aihui Ma, Li Zhang, Guiqin Xu, Qi Ding, Tiantian Jing, Lin Wu, Yun Liu, Zhaojuan Yang, Yongzhong Liu

**Affiliations:** 1 State Key Laboratory of Oncogenes and Related Genes, Shanghai Cancer Institute, Renji Hospital, Shanghai Jiaotong University School of Medicine, Shanghai, China

## Abstract

Hepatitis B virus (HBV) infection is a major factor that contributes to the development of hepatocellular carcinoma (HCC). HBV X protein (HBx) has been shown to accelerate HCC progression by promoting tumour growth and metastasis. In the clinic, carboxyl-terminal truncated HBx (Ct-HBx) proteins are frequently present in HCC tumour tissues, but not in non-tumorous tissues. In this study, we analysed deubiquitinase expression profiles in cells with or without ectopic expression of the Ct-HBx proteins and observed that the expression of ubiquitin specific peptidase 16 (USP16) was substantially inhibited by Ct-HBx proteins. Liver tumour cells with forced down-regulation of USP16 exhibited increased capabilities for colony formation and tumour growth *in vivo*. In addition, USP16 inhibition promoted stem-like properties in tumour cells, as evidenced by their spheroid formation and chemo-responsiveness. Furthermore, ectopic expression of USP16 in tumour cells significantly abrogated the tumour promoting activities of the Ct-HBx proteins (HBxΔ35), leading to decreased tumour cell viability and tumour growth. In human HCCs, USP16 was frequently downregulated, and the decreased expression of USP16 was correlated with high tumour stages and poor differentiation status. Taken together, our study suggests that USP16 downregulation is a critical event in Ct-HBx-mediated promotion of HCC tumorigenicity and malignancy.

Hepatocellular carcinoma (HCC) is the fifth most common malignancy and the third leading cause of cancer-related death worldwide. The prognosis of HCC is approximately 12% survival due to the aggressive metastasis and recurrence of tumours^1^. Chronic hepatitis B virus (HBV) infection is the major crucial factor for the development of HCC^2^,^3^. Emerging evidence indicates that HBV X protein (HBx) has an oncogenic capacity that accelerates HCC progression by promoting cell growth and tumour metastasis^4^,^5^,^6^. Studies have shown that the HBV genome undergoes sequence mutations when integrating into host cells. As a consequence of gene mutation and mRNA editing, HBx mutants, especially carboxyl-terminal truncated HBx (Ct-HBx), have been frequently detected in HCC tumour tissues but are rarely found in adjacent non-tumorous tissues^7^,^8^,^9^. These observations reveal the selective presence of Ct-HBx proteins in the development of HCC, indicating that Ct-HBx proteins may influence biological processes that are critical for HCC initiation and progression. Indeed, mutant HBx proteins increase the threshold of STAT3, NF-κB and PI3K/Akt signals and induce C-jun/MMP10 activation to promote HCC metastasis^10^,^11^,^12^,^13^. Although great advances have been made in understanding the function of Ct-HBx, the mechanisms underlying its pro-tumorigenic activities remain elusive.

Deubiquitinases (DUBs) have emerged as key regulators of tumour development. The human genome encodes approximately 91 active DUBs that regulate homeostasis and multiple cellular processes, including signal transduction, DNA repair and transcriptional activation^14^,^15^,^16^,^17^. USP16 belongs to the ubiquitin-specific protease (USP) family and has the ability to reverse the mono-ubiquitination of H2A^18^. USP16-mediated ubH2A deubiquitination is essential for the activation of lineage-specific gene expression, leading to mouse embryonic stem cell differentiation^19^. In addition, excessive USP16 expression decreases the self-renewal ability of hematopoietic stem cells (HSCs) and the expansion of mammary epithelial cells, neural progenitors and fibroblasts. The function of USP16 may be realized through the regulation of p16^Ink4a^ and p19^Arf^ expression^20^. Therefore, USP16 is involved in the transcriptional regulation that controls cell differentiation, the cell cycle and apoptosis.

In this study, we have revealed a tumour suppressive function of USP16 in liver tumour cells and demonstrated that USP16 downregulation is a key event that is critical for Ct-HBX-mediated pro-tumorigenic activity. We show that USP16 has the capacity to inhibit tumour formation and tumour cell stemness properties. More importantly, USP16 is frequently downregulated in HCC tissues, and its low expression is associated with advanced malignance. Therefore, our present data have provided evidence that USP16 downregulation contributes to the pro-tumorigenic activities of HBx in the development of HCC.

## Results

### Ct-HBx proteins downregulate USP16 expression

**Figure 1:**
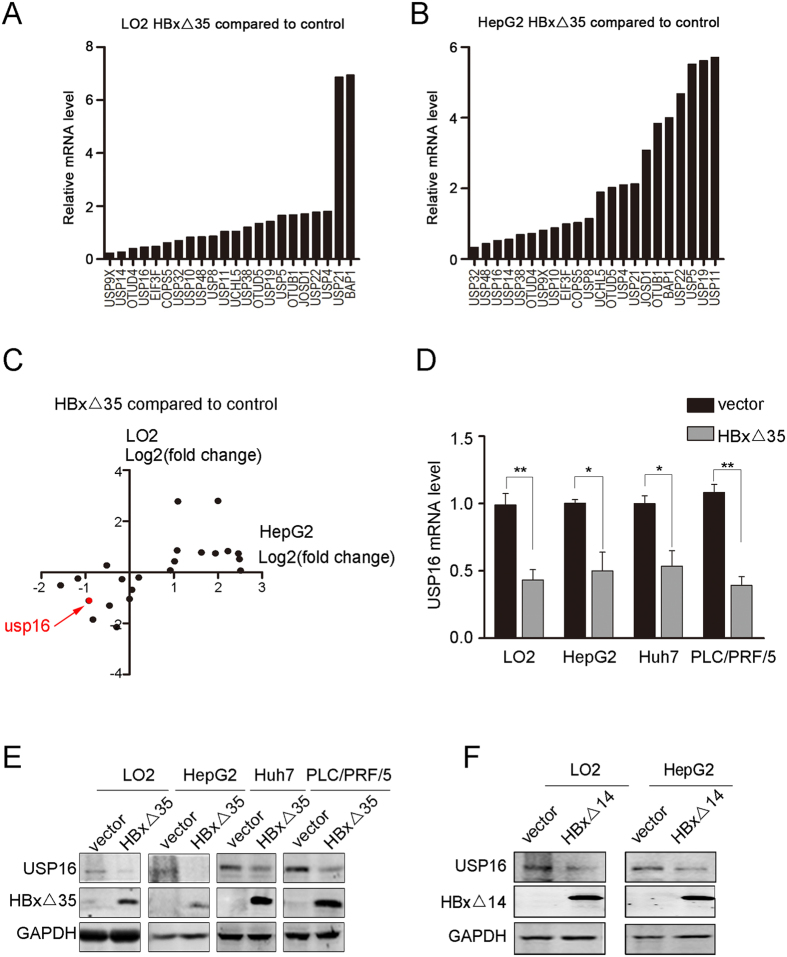
USP16 expression is negatively regulated by COOH-truncated HBx proteins. (**A**,**B**) Comparative analysis of 22 DUB mRNA levels in HBxΔ35-overexpressing LO2 (**A**) and HepG2 (**B**) cells relative to mRNA levels in control cells. (**C**) The log2 values (fold change) for both LO2 and HepG2 were plotted. (**D**,**E**) USP16 expression levels regulated by HBxΔ35 were examined using real-time PCR (**D**) and western blot (**E**) analysis in LO2, HepG2, Huh7 and PLC/PRF/5 cells. (**F**) The expression levels of USP16 regulated by HBxΔ14 were examined by western blot in LO2 and HepG2 cells. The data represent the mean ± S.D, *p < 0.05, **p < 0.01, determined by *t*-test.

To address whether oncogenic Ct-HBx may function by regulating the expression of deubiquitinating enzymes (DUBs), we ectopically expressed a truncated form of HBx, HBxΔ35 (depletion of 35aa from the COOH-terminal)^7^,^21^,^22^, in cells to examine changes at the mRNA level for 22 DUBs. These DUB genes chosen for screening were expressed with relatively high abundances in human liver tissues based on *in silico* EST profile analysis. Changes in the expression patterns caused by HBxΔ35 overexpression in immortalized liver LO2 cells and HepG2 liver tumour cell line were analysed by real-time RT-PCR (Fig. 1A,B). By the cut-off value of fold change ≥2 or ≤0.5, USP16 was identified as a candidate gene downregulated by HBxΔ35 in both cell lines (Fig. 1C). The levels of USP16 mRNA were also decreased in Huh7 and PLC/PRF/5 cells overexpressing HBxΔ35 (Fig. 1D). Furthermore, the protein levels of USP16 were reduced in liver tumour cells expressing the Ct-HBx proteins HBxΔ35 and HBxΔ14^22^ (Fig. 1E,F). Thus, these data indicate that Ct-HBx proteins in liver cancer cells can negatively regulate USP16 expression. In contrast to the COOH-terminal truncated forms, Overexpression of full-length HBx (FL-HBx) did not reduce but slightly increase USP16 expression in HepG2, PLC/PRF/5 tumour cells and liver LO2 cells (Supplementary Fig. 1A,B). Notably, in line with the previous reports showing the inhibitory effects of FL-HBx on cell survival or proliferation in culture^8^,^9^,^13^,^23^,^24^,^25^,^26^, ectopic expression of FL-HBx strongly suppressed the proliferation of the liver cancer cell lines and immortalized liver LO2 cells, and cell lines with stable expression of FL-HBx could not be generated (Supplementary Fig. 2A,B). We thereafter focused on the biological function of Ct-HBx- mediated downregulation of USP16 in liver tumour cells.

### USP16 inhibition enhances tumorigenicity of liver tumour cells

**Figure 2:**
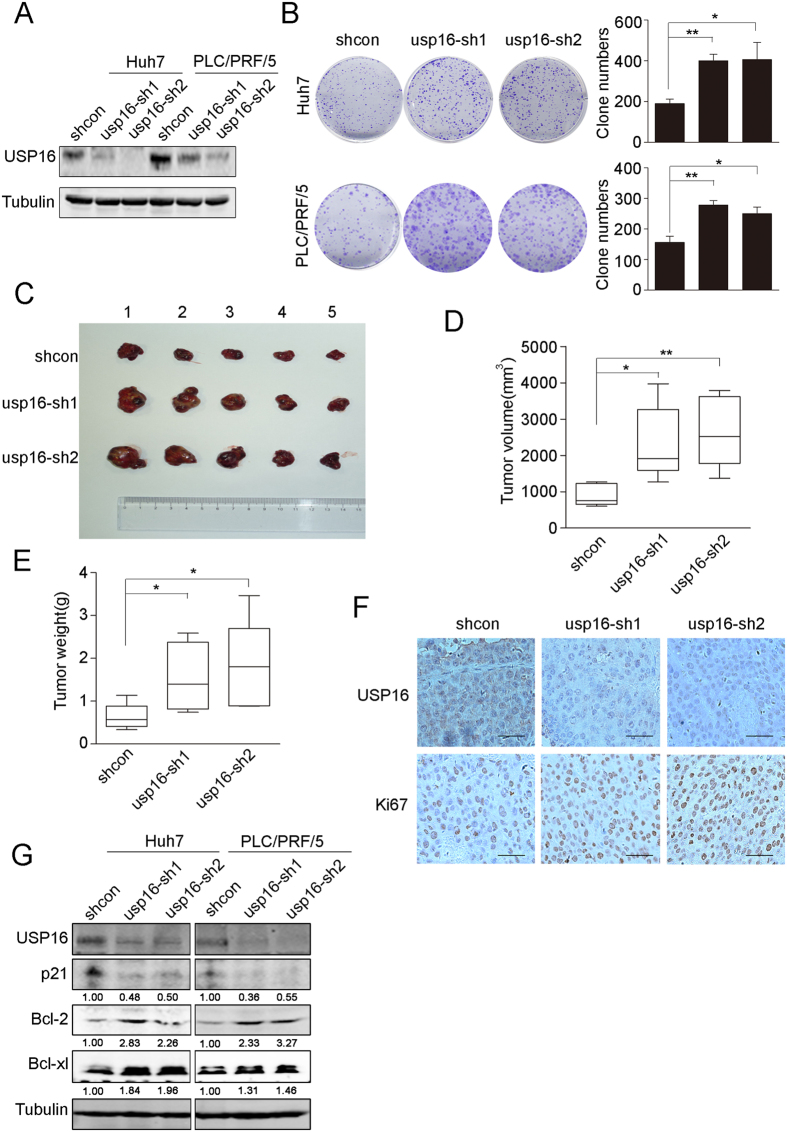
Knockdown of USP16 accelerates the growth of liver tumour cells. (**A**) Down-regulation of USP16 by shRNAs (usp16-sh1 and usp16-sh2) in Huh7 and PLC/PRF/5 cells was confirmed by western blot. (**B**) Images from the colony formation assay are shown (left panel), and the clone numbers for each group were calculated (right panel). (**C**) Tumours formed by Huh7-shcon or Huh7 shUSP16 cells are shown. (**D**,**E**) Tumour volumes (**D**) and tumour weights (**E**) were measured. Box-and-whisker plots are shown. Boxes represent the upper and lower quartiles and median; whiskers show the minimum and maximum data points, *p < 0.05, **p < 0.01, determined by *t*-test. (**F**) Immunostaining analysis of USP16 and Ki67 in subcutaneous tumours (bar, 100 μm). (**G**) Huh7 and PLC/PRF/5 with or without knockdown of USP16 were examined by western blotting to test the expression levels of the indicated proteins.

Ct-HBx has been shown to promote tumour cell proliferation and tumourigenesis^21^,^22^,^27^. To determine whether USP16 downregulation is involved in the oncogenic activities of Ct-HBx proteins, the endogenous USP16 expression in Huh7 and PLC/PRF/5 liver tumour cells was stably silenced by lentivirus-delivered short-hairpin RNAs (shRNAs, usp16-sh1 and usp16-sh2) (Fig. 2A). Depletion of USP16 significantly increased the cell growth rate and inhibited cell anoikis *in vitro* (Fig. 2B and Supplementary Fig. 3A–D). We further examined whether USP16 exerted suppressive activities on tumours grown *in vivo*. The results showed that the tumours derived from Huh7–sh-Usp16 cells grew more vigorously, as evidenced by the tumour volumes and weights, than those formed by control cells (Fig. 2C–E). IHC staining demonstrated that xenograft tumours with USP16 inhibition exhibited increased Ki67 expression in comparison with control tumours (Fig. 2F). Intriguingly, in accordance with the tumour suppressive functions of USP16, USP16 depletion was capable of inhibiting P21 expression but increasing Bcl-XL and Bcl-2 expression in Huh7 and PLC/PRF/5 cells (Fig. 2G). These data were consistent with previous studies showing that decreased P21 expression or increased Bcl-XL/Bcl-2 participated in the regulation of cell proliferation and stem-like properties^28^,^29^. These results indicate that USP16 inhibition by Ct-HBX proteins may provide growth advantages for liver tumour cells *in vivo*.

### Knockdown of USP16 promotes stem-like phenotypes of liver tumour cells

**Figure 3:**
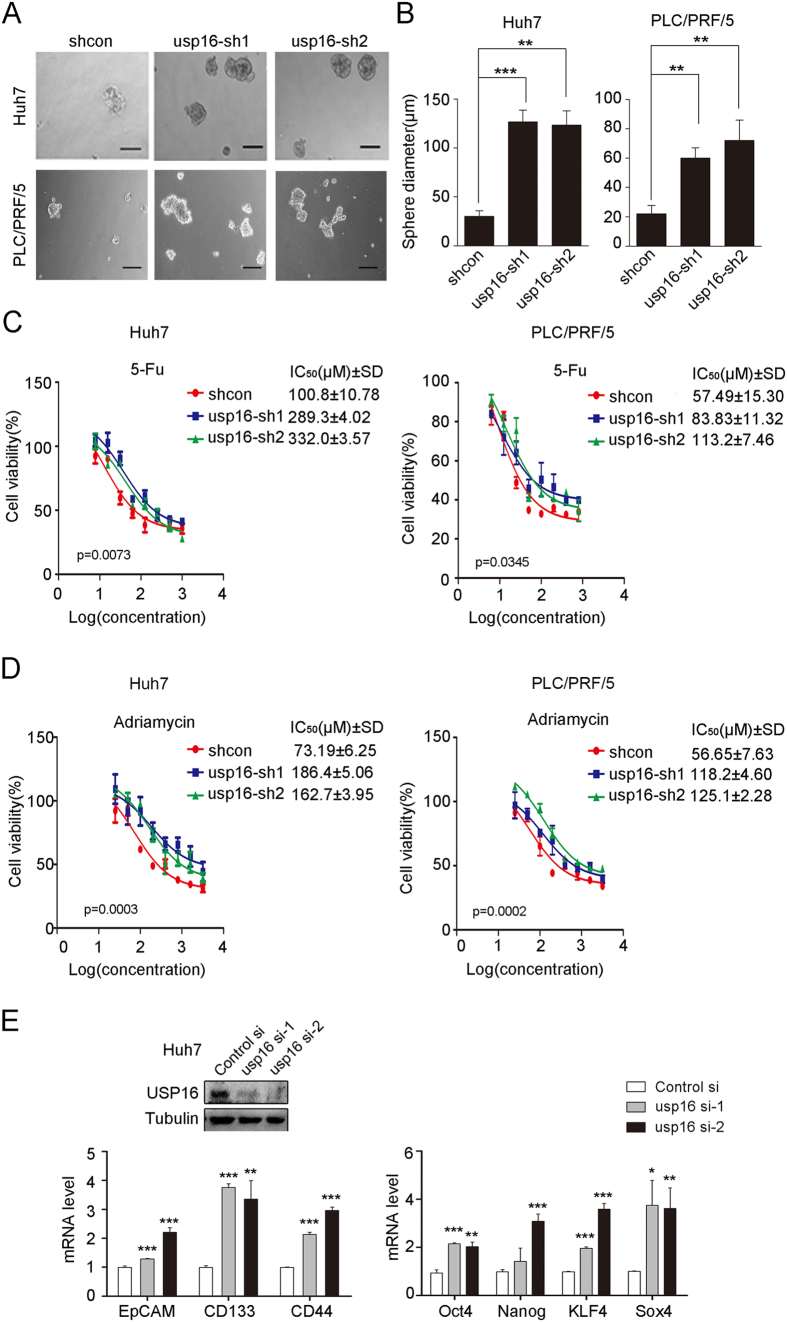
USP16 suppresses the stemness properties of HCC cells. (**A**) Representative images from sphere formation assays after the depletion of USP16 in Huh7 and PLC/PRF/5 cells are shown (bar, 100 μm). (**B**) The diameters of spheres of different groups were measured. The data represent the mean ± S.D, **p < 0.01, ***p < 0.001, determined by *t*-test. (**C**,**D**) Huh7 and PLC/PRF/5 cells with or without USP16 down-regulation were treated with 5-fluorouracil (**C**) and adriamycin (**D**). Cell viabilities were measured three days after drug treatment, and IC50 values were calculated and were tested using one-way ANOVA. The data represent the mean ± S.D. (**E**) The relative mRNA expression levels of the indicated stemness-related genes were analysed using real-time PCR in control and USP16-depleted Huh7 cells. Mean ± S.D are shown, *p < 0.05, **p < 0.01, ***p < 0.001, determined by *t*-test.

Given that USP16 plays a role in regulating embryonic stem cell gene expression, and knocking down USP16 leads to alterations in the expression of P21, Bcl-XL and Bcl-2, we further addressed whether USP16 inhibition could influence the stemness characteristics of liver tumour cells. It was found that knockdown of USP16 significantly increased the spheroid-forming capabilities of liver tumour cells (Fig. 3A,B). In addition, USP16 knockdown decreased the responses of tumour cells to chemo-drugs, as evidenced by alterations in the IC50 values of the tumour cells after exposure to 5-fluorouracil (5-FU) and adriamycin (Fig. 3C,D). Moreover, the up-regulation of some genes related to the stemness of cancer cells, such as EpCAM, CD133, CD44, Oct4, Nanog, KLF4 and Sox4, in USP16-depleted liver cancer cells also suggests a stemness-suppressing role for USP16 (Fig. 3E and Supplementary Fig. 3E.)

### Downregulation of USP16 is required for Ct-HBx to promote tumorigenicity

**Figure 4:**
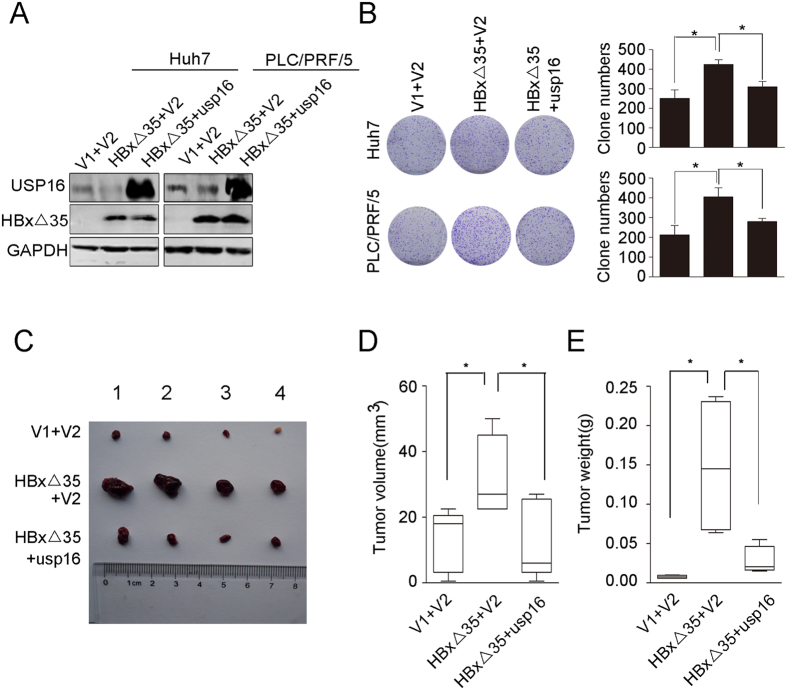
Restoration of USP16 counteracts HBxΔ35-mediated tumorigenesis of HCC cells. (**A**) Western blot of HBxΔ35-Huh7 and HBxΔ35-PLC/PRF/5 cells with or without ectopic expression of USP16, as well as control cells. (V1 indicates the control vector for the HBxΔ35 expression plasmid, and V2 indicates the control vector for the USP16 expression plasmid.) (**B**) Cell proliferation was examined in a colony formation assay. (**C**) HBxΔ35-Huh7 cells with or without ectopic expression of USP16 and control cells were subcutaneously injected into the right flanks of mice. Photographs of the tumours are shown. (**D,E**) Tumour volumes (**D**) and tumour weights (**E**) were measured. Box-and-whisker plots are shown. Boxes represent the upper and lower quartiles and median; whiskers indicate the minimum and maximum data points. The data represent the mean ± S.D, *p < 0.05, determined by *t*-test.

The aforementioned results revealed the tumour-suppressive functions of USP16, which could be downregulated by Ct-HBx in liver tumour cells. To test whether negative regulation of USP16 expression is required for pro-oncogenic activities of Ct-HBx, we employed lentiviruses with or without a USP16-expressing cassette and infected Huh7 and PLC/PRF/5 cells that stably expressed HBxΔ35 (Fig. 4A). Colony formation and MTT assays showed that USP16 overexpression counteracted HBxΔ35-mediated enhancement of cell proliferation (Fig. 4B and Supplementary Fig. 4A,B). Furthermore, HBxΔ35-mediated protection of liver tumour cells from anoikis was significantly suppressed by exogenous USP16 expression (Supplementary Fig. 4C,D). We further corroborated the suppressive effects of USP16 on the oncogenic activities of Ct-HBx *in vivo* by injecting HBxΔ35-transduced Huh7 cells and HBxΔ35, USP16-cotranduced Huh7 cells into nude mice and measuring the sizes and weights of the xenograft tumours after 3 weeks. The results showed that tumours that developed from HBxΔ35-transfected cells were significantly larger and heavier than the tumours derived from control cells, whereas the tumours formed by HBxΔ35, USP16-cotransfected cells were markedly suppressed in comparison with HBxΔ35-transduced cells (Fig. 4C–E), indicating that the oncogenic effects of the HBxΔ35 protein were abrogated by the restoration of USP16 expression. Collectively, our data demonstrate that the decreased expression of USP16 is essential for the pro-tumorigenic activities of Ct-HBx.

### Ct-HBx-driven stem-like properties are inhibited by forced USP16 expression in liver tumour cells

**Figure 5:**
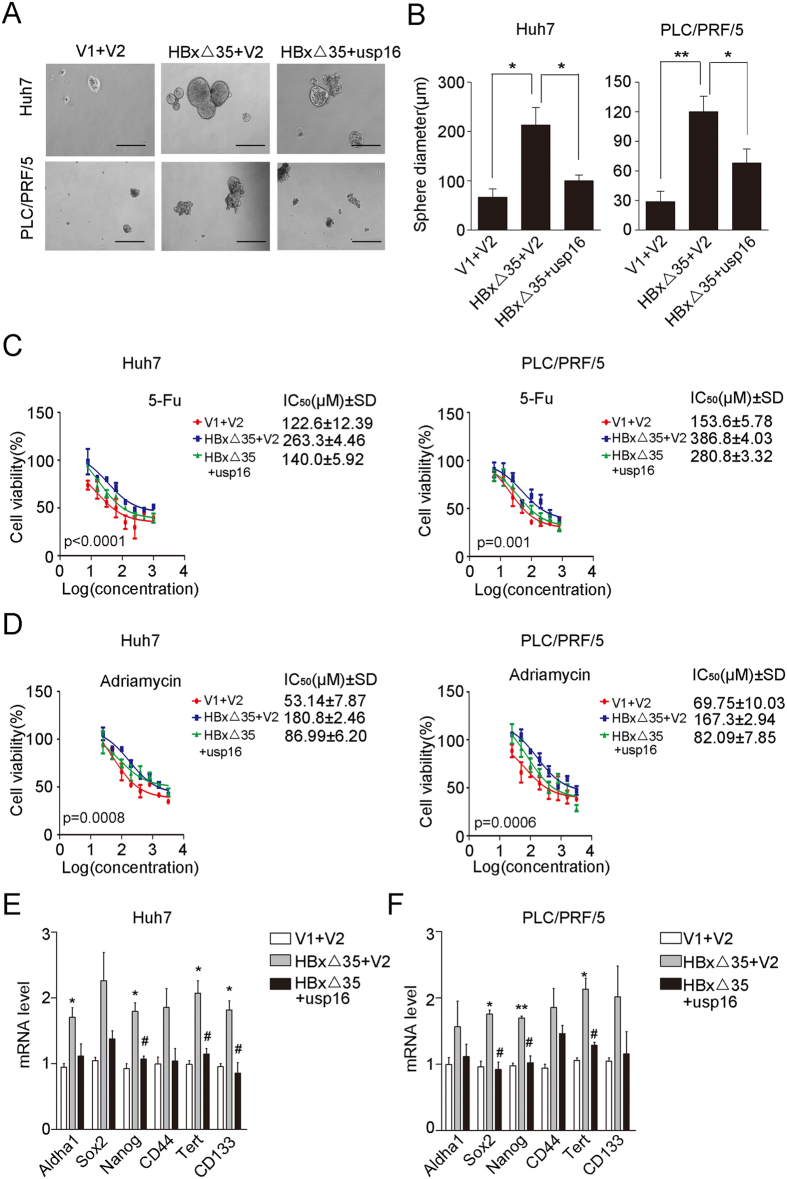
Ectopic USP16 expression attenuates HBxΔ35-driven stem-like properties of HCC cells. (**A**,**B**) HBxΔ35-Huh7 and HBxΔ35-PLC/PRF/5 cells with or without ectopic USP16 expression and their control cells were cultured on ultra-low adherent wells for sphere formation. Representative photographs are shown (**A**) (bar, 100 μm). Additionally, the diameters of spheres for different groups were measured (**B**). The data represent the mean ± S.D, *p < 0.05, **p < 0.01, determined by *t*-test. (**C**,**D**) HBxΔ35-Huh7 and HBxΔ35-PLC/PRF/5 cells with or without ectopic expression of USP16 and their control cells were treated with 5-fluorouracil (**C**) and adriamycin (**D**), and cell viabilities were measured. IC50 values were calculated and were tested using one-way ANOVA. (**E**,**F**) Real-time RT–PCR was performed to analyse the expression of stemness-related genes in Huh7 (**E**) and PLC/PRF/5 cells (**F**). *compared to V1 + V2; ^#^compared to HBxΔ35 + V2, *p < 0.05, **p < 0.01, ^#^p < 0.05, determined by *t*-test.

It has been reported that the oncogenic roles of HBx proteins are largely attributable to their regulation of stemness in HCC cells^27^,^30^,^31^. To corroborate whether the regulation of stem-like properties by Ct-HBx is related to USP16 downregulation, we comparatively analysed the stem-like properties of liver tumour cells with ectopic HBxΔ35 expression in comparison with cells co-expressing HBxΔ35 and USP16. The results showed that USP16 overexpression counteracted the HBxΔ35-mediated enrichment of stem-like cells, as evident by the alterations in spheroid formation (Fig. 5A,B) and the chemo-responses in the cells examined (Fig. 5C,D). More importantly, we also found that USP16 expression significantly attenuated the HBxΔ35-mediated upregulation of mRNA levels of stem cell-associated genes such as Sox2, Nanog and CD44 (Fig. 5E,F). Thus, these data indicate that the downregulation of USP16 by HBxΔ35 may contribute to the stemness properties of liver tumour cells.

### USP16 is downregulated and negatively associated with the malignant phenotypes of HCC

**Figure 6:**
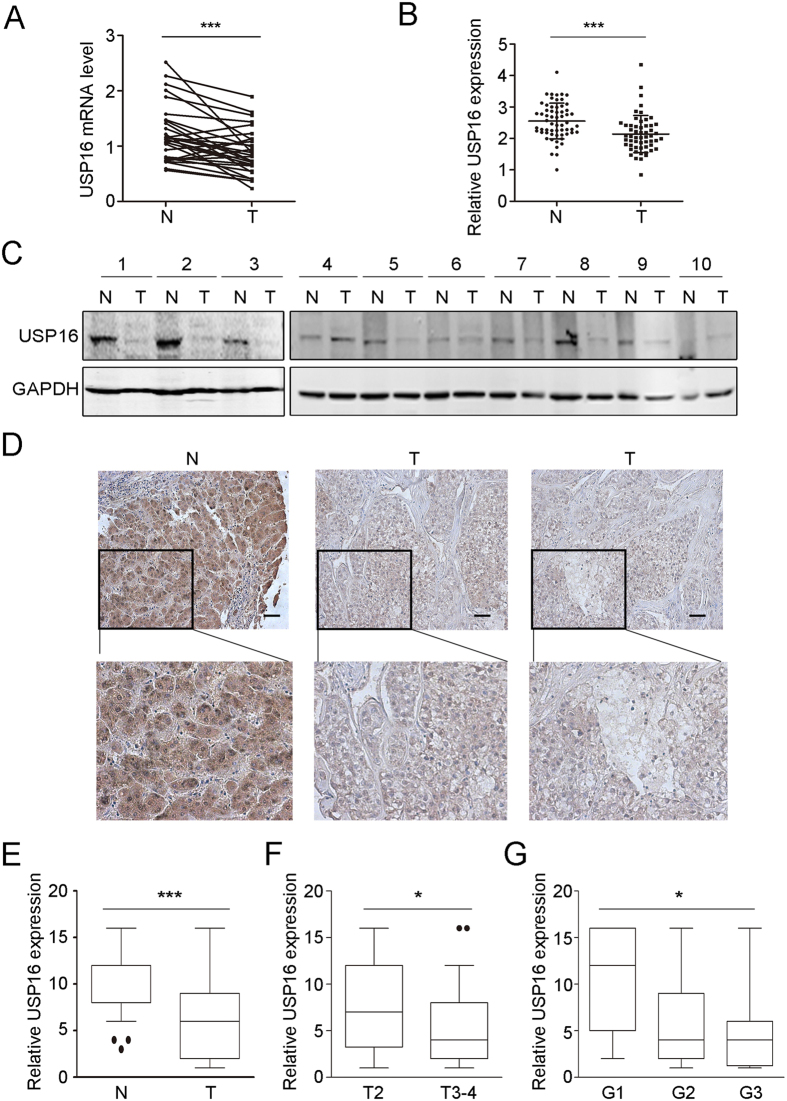
USP16 is down-regulated in human HCC tissues and correlates with tumour stages. (**A**) USP16 mRNA levels in 26 HCC and paired non-tumour tissues were analysed. P values were calculated using a paired *t*-test, ***p < 0.001. (**B**) Expression levels of USP16 in non-tumoral and liver tumour tissues from the GSE14323 dataset. P values were calculated using the Wilcoxon signed rank test, ***p < 0.001. (**C**) Western blot analysis of USP16 expression in 10 cases of paired HCC samples. (**D**) Representative images of USP16 expression in non-tumour and liver tumour tissues (bar, 100 μm). (**E**) Box-and whisker plots of the staining quantified by morphometry. (**F**,**G**) Clinical correlation of USP16 expression with T stages (**F**) and differentiation status (**G**). Box-and-whisker plots are shown. Boxes represent the upper and lower quartiles and median; whiskers indicate the minimum and maximum data points, *p < 0.05, **p < 0.01, ***p < 0.001, determined by Wilcoxon signed rank test.

Chronic HBV infection is one of the most important etiological factors contributing to the development of HCC, and the majority of HBx-positive HCCs express Ct-HBx proteins^32^,^33^,^34^. Indeed, our results showed that carboxyl-terminal deletions of HBx were selectively present in 7 of 12 cases of human HCC tumour tissues but not in the paired non-tumour tissues (Supplementary Fig. 5). We further examined the expression levels of USP16 in the paired HCC tissues and found that the mRNA levels of USP16 were significantly decreased in tumour tissues compared with those in the adjacent non-tumour specimens (Fig. 6A). Moreover, *in silico* analysis of the HCC GSE14323 dataset, which includes a relatively large number of HCC samples, confirmed the downregulation of USP16 expression in tumour samples (Fig. 6B). In addition, we found that the protein levels of USP16 were decreased in 6 of 10 cases in paired tumour and non-tumour HCC tissues (Fig. 6C). Finally, we assessed the expression status of USP16 in 100 HCC patients by immunohistochemistry staining to evaluate the association of USP16 with malignant tumour phenotypes. Interestingly, USP16 was expressed in both the nucleus and cytoplasm and was markedly decreased in tumour tissues (Fig. 6D,E). Correlation analysis of the expression of USP16 and clinicopathological parameters showed that low levels of USP16 were associated with high tumour stages and poor differentiation (Fig. 6F,G). These results indicate that USP16 expression is down-regulated in HCCs and negatively associated with malignant phenotypes.

## Discussion

HCC is one of the most common cancers worldwide, and HBV-associated carcinogenesis is a major factor in pathopoiesis, especially in China^35^. HBx frequently undergoes deletion during HBV integration, generating HBx mutants that encode Ct-HBx proteins. It is reported that 46% of human HCC tissues contain Ct-HBx DNA, and the presence of Ct-HBx in HCC tissues is associated with venous invasion^13^. *In vivo* tumorigenesis experiments have shown that Ct-HBx induces HCC more rapidly than FL-HBx^36^. A clinical trial suggests that Ct-HBx expression is correlated to decreased recurrence-free survival (RFS) in HCC patients without antiviral treatment^37^. Moreover, human APOBEC3 can edit the HBx gene and a premature stop codon generated at amino acid 120 results in the synthesis of HBxΔ35, which enhances the colony formatting ability of neoplastic cells^7^. Ct-HBx is known to promote tumour cell proliferation, migration and invasion^7^,^13^,^38^, the roles of FL-HBx, however, have been controversially reported in literature. While FL-HBx has been considered to possess pro-apoptotic or cytotoxic activities, which can be abrogated by the COOH-terminal depletion^8^,^9^,^13^,^23^,^24^,^25^,^26^, some previous data suggest that FL-HBx is able to promote liver cell transformation and proliferation^39^,^40^. The facts to reconcile these apparent discrepancies remain unknown. Previous evidence has shown that certain mutations of HBx cDNA sequence may affect the functions of HBx and probablely eliminate the deleterious effects of FL-HBX proteins to cell proliferation^41^,^42^,^43^,^44^,^45^,^46^. It is therefore questionable whether different types of HBx with distinct function were used in the studies that brought out the contradictory results. Another possibility, which seems less likely to occur but cannot be definitely excluded, is methodological differences, including the variations in cell context as well as in the ways of gene transduction and selection. Nevertheless, our present data confirmed the functional differences between FL-HBx and CT-HBx proteins. Sustained expression of Ct-HBx obviously accelerated cellular proliferation, whereas cells expressing FL-HBx were not competent to proliferate in culture. Interestingly, we also found that the two forms of HBx proteins regulated USP16 expression in opposite ways and the down-regulation of USP16 was specific to the Ct-HBx protein. Restoration of USP16 in Ct-HBx expression cells alleviated the oncogenic effects of Ct-HBx, suggesting the important roles of USP16 downregulation in Ct-HBx signalling. Notably, the difficulties in the establishment of cell lines with sustained FL-HBx-expression did not allow us to set up control to systemically examine the functions of USP16 in Ct- and Fl-HBx protein-related biological effects. The involvement of USP16 in the activities of FL-HBx needs further investigation.

Emerging evidence has shown that deregulation of DUBs is involved in tumorigenesis and cancer progression^47^,^48^,^49^. In this study, we explored whether the expression of DUBs could be regulated by Ct-HBx using two Ct-HBx mutants, HBxΔ14 and HBxΔ35. After screening, we found that USP16 expression was concomitantly downregulated by these two mutants. Consistently, clinical data present in the study also demonstrate decreased levels of USP16 in HCC tumours compared with non-tumour tissues. The results of *in vivo* tumour formation further showed that knockdown of USP16 could significantly enhance the tumorigenicity of tumour cells. In accordance with this observation, we also found that knockdown of USP16 decreased P21 but increased Bcl-XL/Bcl-2 expression in liver tumour cells. These data collectively indicate a suppressive role for USP16 in the development of HCC. Usp16 regulates H2A deubiquitination and modulates self-renewal and differentiation in different types of cells. Excessive USP16 expression causes defects in the self-renewal of hematopoietic stem cells and the propagation of other types of cells^20^. On the contrary, ESCs with Usp16 deletion are defective in differentiation because of the ubH2A-mediated inhibition of lineage-specific genes^19^. Moreover, these observations were also noted for hematopoietic stem cells^50^. In accordance with these findings, we found that knockdown of USP16 significantly increased spheroid-forming capability and decreased the vulnerability of liver tumour cells to 5-FU and adriamycin treatments. These results suggest that, in addition to the role of USP16 in regulating the properties of adult progenitor cells, USP16 may have exert deleterious effects on tumour stem cells. Furthermore, the enhancement of stem cell properties and tumorigenicity in liver tumour cells by Ct-HBx proteins can be abrogated by USP16 overexpression, indicating that downregulation of USP16 is critical for Ct-HBx-related pro-stemness activities. Notably, as a limitation of the present study, and mostly due to inadequate source of clinical samples, the relationship between Ct-HBx presence and USP16 expression has not been analysed in a large collection of HCC specimens. Apparently, the exploration of clinical relationship between Ct-HBx and USP16 expression is important and warrants future studies.

In conclusion, our study suggests that USP16 is negatively regulated by Ct-HBx and plays a critical role in the pro-tumorigenicity of Ct-HBx proteins. Clinically, the downregulation of USP16 occurs frequently in HCC tissues and correlates with clinical stages and tumour cell differentiation. Therefore, this study provides new insights into the mechanism underlying the promotion of HCC development and progression by Ct-HBx.

## Methods

### Ethics statement

All studies using human materials were approved by the Ethical Review Committee of Renji Hospital, School of Medicine, Shanghai JiaoTong University, and the written informed consent forms were obtained from all patients for research. All mouse studies were approved by the Animal Care and Use Committee of Shanghai Cancer Institute, Renji Hospital, School of Medicine, Shanghai JiaoTong University. All the methods in this study were performed in accordance with the approved guidelines and regulations.

### Cell lines and clinical specimens

The liver tumour cell lines HepG2, Huh7, and PLC/PRF/5 and the human embryonic kidney 293T (HEK293T) cell line were provided by the American Type Culture Collection (ATCC). The immortalized liver cell line LO2 was obtained from the Shanghai Cancer Institute. All cells were maintained in DMEM with high glucose (GIBCO) supplemented with 10% foetal bovine serum, penicillin (100 U/ml) and streptomycin (100 μg/ml). Cells were incubated at 37 °C in a humidified incubator under 5% CO_2_. Fresh human tumour and paired adjacent non-tumour liver tissues to analyse USP16 mRNA and protein expression levels were obtained from the Department of Transplantation and Hepatic Surgery, Ren Ji Hospital, School of Medicine, Shanghai JiaoTong University. Human HCC tissue microarrays were commercially provided for the analysis of USP16 expression.

### Cancer stem-like cell culture and lentivirus infection

To culture stem-like cells, the serum-free medium was composed of DMEM/F12, 10 μg/ml bFGF, 20 μg/ml EGF and B27 supplement. Cells were suspended to form sphere-like cell aggregates. Full-length and COOH-terminal truncated forms of HBx (HBxΔ35 and HBxΔ14), that were from non-tumoral and liver cancer tissues respectively, were cloned into pLVX-IRES-zsGreen vector; USP16 was cloned into pLVX-IRES-puro and pLVX-IRES-zsGreen vectors. shRNAs targeting the USP16 expression sequence were cloned into the pPRIME plasmid. Lentiviral particles were constructed after transfecting the vector with the packaging plasmid psPAX2 and envelope plasmid pMD2G into HEK293T cells using Lipofectamine 2000 transfection reagent. The indicated cells were infected with lentivirus plus Polybrene. siRNAs used were as follows: usp16 si-1: TAGTGAATGTGGAATGGAA; usp16 si-2: CAGTGAATATGAAGCTGAA

### *In vivo* tumour formation assay

For *in vivo* tumour formation, 10^6^ cells were subcutaneously implanted into nude BALB/c mice (5 to 6 weeks of age). Tumour size was serially measured using callipers. Tumour-bearing mice were euthanized, and the tumours were removed for further study.

### Immunohistochemical staining

Immunohistochemical analysis of HCC tissue microarrays using an anti-USP16 antibody (Santa Cruz Biotechnology) was performed as previously described^49^. To analyse the tissue microarrays, all IHC staining was assessed independently by two pathologists and scored according to the ratio and intensity of positive staining. Briefly, the ratio was graded from 1 to 4 based on the percentage of positively stained cells (1, 0–15%; 2, 16–50%; 3, 51–75%; 4, 76–100%). The intensity was graded from 1 to 4 (1, no staining; 2, weak staining; 3, moderate staining; 4, strong staining). A final score from 1 to 16 was applied based on the multiplying ratio and intensity. For each sample, the expression level was indicated as negative (≤8) or positive (>8).

### Statistical analysis

Statistical analyses were applied with SPSS 21.0 software and GraphPad Prism 5 software. The statistical significance of USP16 expression in tumour and non-tumorous tissues was determined using the Wilcoxon matched pair’s signed-rank test. The relationship between clinicopathological parameters and USP16 expression was analysed using the Wilcoxon signed-rank test. The data are presented as the mean ± S.D. from at least three independent experiments. All *in vitro* and *in vivo* experiments were assessed using Student’s *t*-test or a one way analysis of variance (ANOVA) test. A p-value of less than 0.05 was considered statistically significant.

Detailed Materials and Methods are available in the Supplementary data.

## Additional Information

**How to cite this article**: Qian, Y. *et al.* USP16 Downregulation by Carboxyl-terminal Truncated HBx Promotes the Growth of Hepatocellular Carcinoma Cells. *Sci. Rep.* **6**, 33039; doi: 10.1038/srep33039 (2016).

## Supplementary Material

Supplementary Information
