# Supplementary material for: USP16 Downregulation by Carboxyl-terminal Truncated HBx Promotes the Growth of Hepatocellular Carcinoma Cells
Source: Sci Rep. 2016 Sep 16;6:33039. doi: 10.1038/srep33039 (PMC5025738; doi:10.1038/srep33039)
Supplement: Supplementary Information [file srep33039-s1.pdf]

USP16 Downregulation by Carboxyl-Terminal Truncated HBx promotes Growth of Hepatocellular Carcinoma Cells

Yu Qian[1, #], Boshi Wang[1, #], Aihui Ma[1], Li Zhang[1], Guiqin Xu[1], Qi Ding[1],Tiantian Jing[1], Lin Wu[1], Yun Liu[1], Zhaojuan Yang[1] and Yongzhong Liu[1*]

Author Information: State Key Laboratory of Oncogenes and Related Genes, Shanghai Cancer Institute, Renji Hospital, Shanghai Jiaotong University School of Medicine, Shanghai, China.

Corresponding author:

Yongzhong Liu Ph.D.

Principal Investigator

State Key Laboratory of Oncogenes and Related Genes,

Shanghai Cancer Institute,

Renji Hospital, Shanghai Jiaotong University School of Medicine.

Wenxuan Medical Building, 800 Dongchuan Road, Shanghai, China 200240

Email: liuyzg@shsci.org, Tel: 86-21-3420-6283, Fax: 86-21-3420-6283

Author contributions:

Y.Q., B.W., Y.L. designed the research, analysed data and wrote the manuscript; Y.Q., B.W., A.M., L.Z., G.X., Z.Y., Y.L., Q.D., T.J., L.W performed the experiments.

[#] These authors contributed equally to this work.

Supplementary Materials and Methods

Quantitative Real-time PCR (qRT-PCR)

Total RNA from tissue samples and cells were extracted using RNAiso Plus kit (Takara).For qRT-PCR, cDNA preparation was performed by PrimeScript RT regent kit (Takara) and used for templates in SYBR-Green method. All reactions were performed using ABI7500 instrument. The primers used for detecting mRNA levels were listed in Table S1.

Western blot

The total proteins of tissue samples or tumour cell lines were extracted using RIPA lysis buffer (Thermo Scientific) containing cocktail of proteinase inhibitors and phosphatase inhibitors according to the protocol. For western blot assay, 60μg of total protein lysates were subjected to 12% SDS-PAGE, transferred on to nitrocellulose membranes and then blocked with 5% non-fat milk and incubated with primary antibodies. The protein was detected using enhanced chemiluminescence reagents. Anti-USP16 (14055-1-AP) and anti-Bcl-xl (10783-1-AP) were from Proteintech. Anti-tubulin (sc-69969), anti-GAPDH (sc-25778), anti-p21 (sc-53870) and anti-HBx (sc-57760) were from Santa Cruz Co., anti-Bcl2 (1017) was from Epitomics.

Cell proliferation and colony formation assays

For cell proliferation assay, cells were seeded in 96-well plate at a density of 2000 cells per well. Then at the indicated time points, 20μl of MTT reagent was added to each well. After 3h of incubation at 37℃, the absorbance was read at 590nm using microplate reader. For the colony formation assay, 1000-5000 cells were plated in 6-well plate and incubated at 37℃ for 1 week. The cells were fixed and stained with 1% crystal violet.

TUNEL assay

TdT-mediated dUTP Nick-End Labeling (TUNEL) assay was performed using One Step TUNEL Apoptosis Assay Kit (Beyotime Biotechnology) according to the manufacture's recommendation. Briefly, cells in suspension were centrifuged and harvested. Then cells were fixed and washed. After incubated with 0.1% Triton X-100, cells were staining with TUNEL reagent and counterstained with DAPI. TUNEL-positive cells were detected under the microscope.

Cell anoikis analysis

For cell anoikis analysis, indicated cells were placed in ultra-low attachment 6-well plates for 2 days. Aggregated cells were harvested and digested, then washed with PBS and binding buffer. Apoptotic cells were detected using Annexin V/7-AAD staining (BD Biosciences, USA) with FACS Calibur flow cytometer (BD).

Table S1. Primer sequences

Primer name	Sequence (5’ to 3’)
Primers for qRT-PCR	
homo-SYBR-GAPDH-F	ACCACAGTCCATGCCATCA
homo-SYBR-GAPDH-R	TCCACCACCCTGTTGCTGTA
homo-SYBR-COPS5-F	GCCCTGGACTAAGGATCACC
homo-SYBR-COPS5-R	CTTCCAAGTTGCCTCCCGAT
homo-SYBR-USP38-F	AGAACTGGTTTTTAATCGACTTTGG
homo-SYBR-USP38-R	CGCCTCTGGAGAATGCTGAA
homo-SYBR-USP19-F	GCCTATGTACTCTTCTACCGCC
homo-SYBR-USP19-R	CCAAATCCGGGAAGCCTGG
homo-SYBR-USP5-F	TTCTCCTCTCCGACATCGCC
homo-SYBR-USP5-R	TTGCCCGTGTAGTAGACAGC
homo-SYBR-JOSD1-F	CGCCCTCAATAACGTCTTCCA
homo-SYBR-JOSD1-R	GCATGCTCTTCTTGTGAGGTG
homo-SYBR-USP4-F	TGAAACACGGCTCTGGAACA
homo-SYBR-USP4-R	ACTAGCACCTGACCCTGGTA
homo-SYBR-USP14-F	AGGAGGAACGCTAAAGGATGA
homo-SYBR-USP14-R	TTGGCTGAGGGTTCTTCTGG
homo-SYBR-USP9X-F	AGAGCAAAGAGCCAGATGACC
homo-SYBR-USP9X-R	GCTGTCCATGCACATGGTTATT
homo-SYBR-EIF3F-F	CTGAACGCATCGGAGTTGAC
homo-SYBR-EIF3F-R	GCTGATGCCCCTCCTACTTG
homo-SYBR-USP48-F	ACCCAAGTGTGGCAAAGGAA
homo-SYBR-USP48-R	AGCTCTTGAAGAAAGGCTGGA
homo-SYBR-USP11-F	GGATAGAGAGCCACAGCACG
homo-SYBR-USP11-R	CACAAGGAACCAGCTTTCGC
homo-SYBR-USP10-F	GCCTTGTACGTCAACACCCA
homo-SYBR-USP10-R	ATCTCCAAGAGCTTGTCGGG
homo-SYBR-USP21-F	GTGCGTATACTGCTCCCCAC

homo-SYBR-USP21-R	ACAGGCTGGACCCACAATC
homo-SYBR-USP16-F	GGACAACTGGAGTGTATGGTGT
homo-SYBR-USP16-R	ATCTTTCTCTGGCTTTGGAGTTG
homo-SYBR-BAP1-F	CAAATGGATCGAAGAGCGCC
homo-SYBR-BAP1-R	GGTATCAGCTGGTGGGCAAA
homo-SYBR-OTUD5-F	CCAGTACAGCACAGAACCCA
homo-SYBR-OTUD5-R	CCAATGGTGGCCTTGTTAGG
homo-SYBR-USP22-F	AACTGCACCATAGGTCTGCG
homo-SYBR-USP22-R	AAGAAGTCCCGCAGAAGTGG
homo-SYBR-OTUD4-F	ATTGTAACGCCGGAGTGGG
homo-SYBR-OTUD4-R	AGACTGAGAGTGCAATACCTCTT
homo-SYBR-USP32-F	CGAGATCAGGCTCCGACC
homo-SYBR-USP32-R	CATCTGTAACTCTCCTCAGCG
homo-SYBR-OTUB1-F	CAATTGGCAACCCGGAAGC
homo-SYBR-OTUB1-R	TTAACACCTTCGGAGTCGCTG
homo-SYBR-UCHL5-F	GGATACAAAAGTACAGTGAAGGTGA
homo-SYBR-UCHL5-R	ATCCATGGGTTCCTCTGCAA
homo-SYBR-USP8-F	TCCGCTGTGAACGATGAACG
homo-SYBR-USP8-R	AGTGCTTCTTTCCTTTAGGAGTTG
homo-SYBR -TERT-F	CGGAAGAGTGTCTGGAGCAA
Homo-SYBR-TERT-R	GGATGAAGCGGAGTCTGGA
homo-SYBR-ALDHA1-F	ACCTGTCCTACTCACCGATT
homo-SYBR-ALDHA1-R	AAGCATCCATAGTACGCCAC
homo-SYBR-EpCAM-F	CGCAGCTCAGGAAGAATGTG
homo-SYBR- EpCAM-R	TGAAGTACACTGGCATTGACG
homo-SYBR- CD133-F-1	TCCACAGAAATTTACCTACATTGG
homo-SYBR- CD133-R-1	CAGCAGAGAGCAGATGACCA
homo-SYBR- CD133-F-2	GATTAAGTCCATGGCAACAGCG
homo-SYBR- CD133-R-2	GCTGGTCAGACTGCTGCTAAGC

homo-SYBR-CD44-F-1	ACGGAAGAAACAGCTACCCAG
homo-SYBR-CD44-R-1	GAGGCTGCAGCTGTCCC
homo-SYBR-CD44-F-2	CTGCAGGTATGGGTTCATAG
homo-SYBR-CD44-R-2	ATATGTGTCATACTGGGAGGTG
homo-SYBR-Oct4-F	GGAAGGTATTCAGCCAAACGAC
homo-SYBR-Oct4-R	AGTCGCTGCTTGATCGCTTG
homo-SYBR-NANOG-F	GTCCCGGTCAAGAAACAGAA
homo-SYBR-NANOG-R	TGCGTCACACCATTGCTATT
homo-SYBR-KLF4-F	ACGATCGTGGCCCCGGAAAAGGACC
homo-SYBR-KLF4-R	TGATTGTAGTGCTTTCTGGCTGGGCTCC
homo-SYBR-SOX4-F	CCTCAAGCACATGGCTGACT
homo-SYBR-SOX4-R	CACCGACCTTGTCTCCCTTC
homo-SYBR-SOX2-F	ATGGGTTCGGTGGTCAAGT
homo-SYBR-SOX2-R	ATGTGTGAGAGGGGCAGTGT
Primers for detecting HBx status	
XF1	CAGCTTGTTTTGCTCGCAGC
XR1	GCAGATGAGAAGGCACAGAC
XF2	TCACCTCTGCACGTAGCATGG
XR5	GGCAGAGGTGAAAAAGTTGC

Supplementary Figures

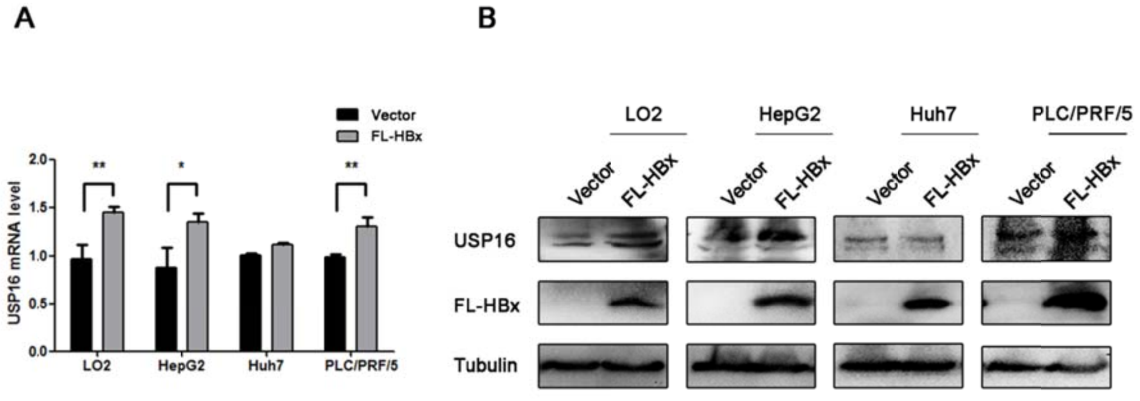

Supplementary Fig.1 Full-length HBx does not decrease USP16. (**a-b**) The LO2, HepG2, Huh7 and PLC/PRF/5 cells were infected with the lentivirus expressing the full-length HBx. Six days after infection, cells were subjected to analyses with real-time PCR (**a**) and western blotting (**b**), *p<0.05, **p<0.01, determined by *t*-test.

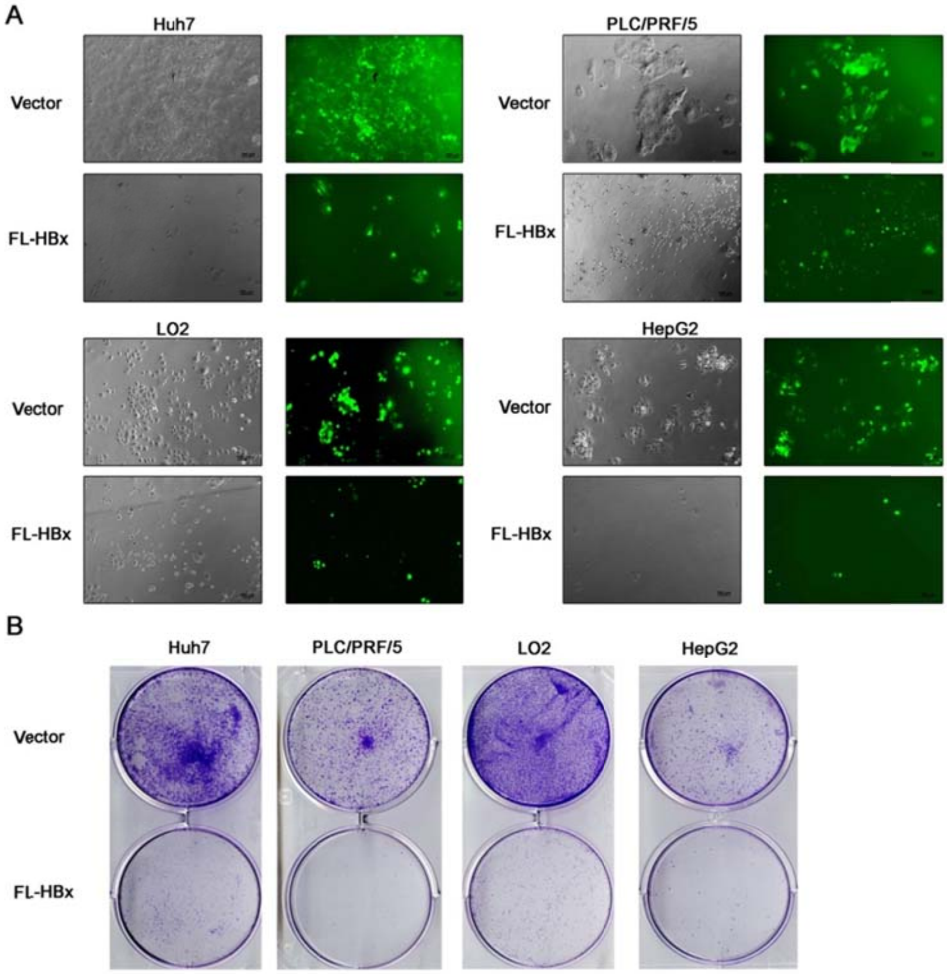

Supplementary Fig. 2 Full length HBx inhibits cell growth. (a-b) The phase contrast and fluorescent microscopic images of the indicated cells lentivirally expressing the control and FL-HBx **(a)** and cell colony formation **(b)** are shown.

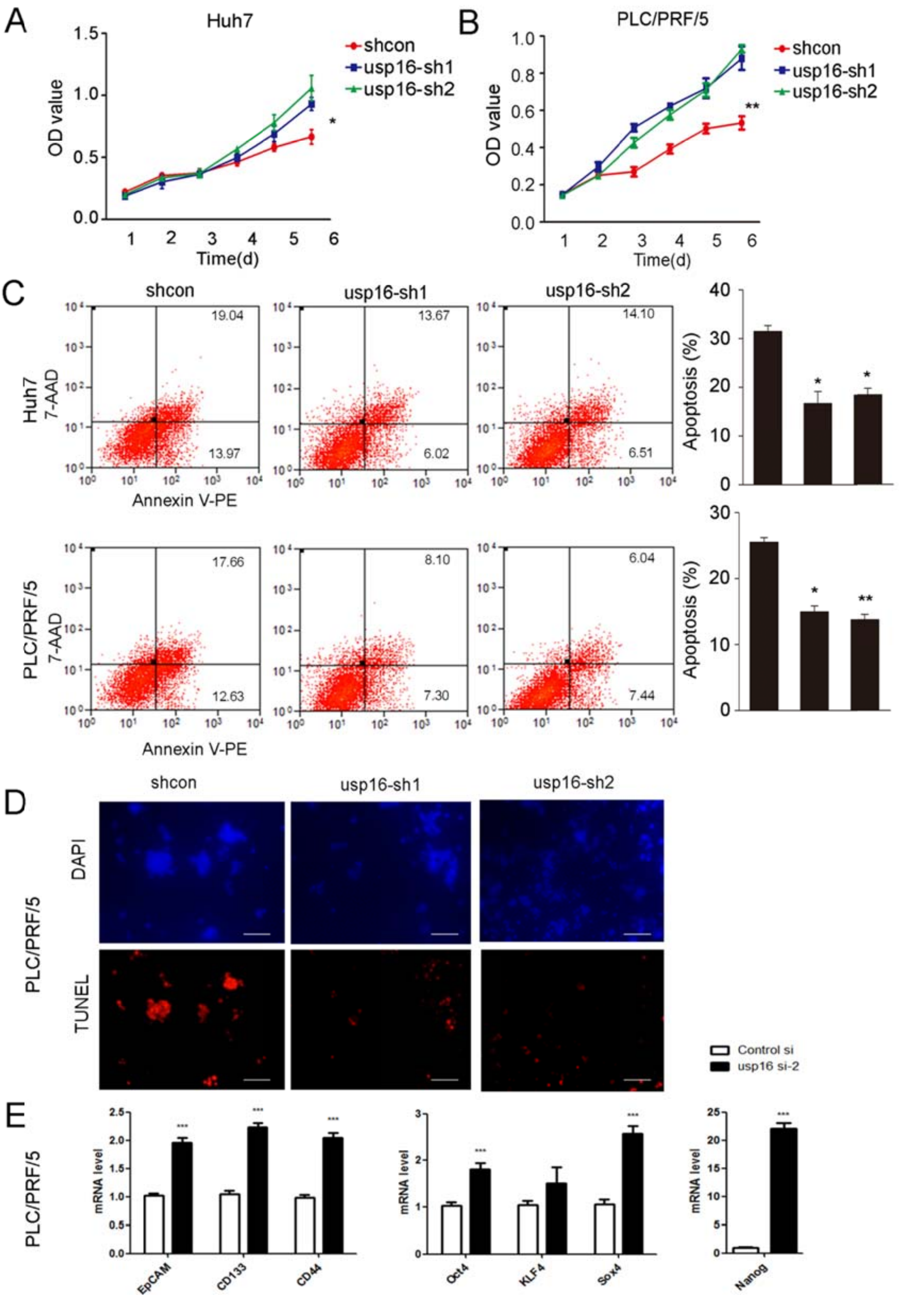

A
Huh7
OD value
Time(d)
shcon
usp16-sh1
usp16-sh2
B
PLC/PRF/5
OD value
Time(d)
shcon
usp16-sh1
usp16-sh2
C
shcon
usp16-sh1
usp16-sh2
Huh7
7-AAD
Annexin V-PE
19.04
13.97
13.67
6.02
14.10
6.51
Apoptosis (%)
PLC/PRF/5
7-AAD
17.66
12.63
8.10
7.30
6.04
7.44
Annexin V-PE
Apoptosis (%)
D
shcon
usp16-sh1
usp16-sh2
PLC/PRF/5
DAPI
TUNEL
Control si
usp16 si-2
E
PLC/PRF/5
mRNA level
EpCAM
CD133
CD44
mRNA level
Oct4
KLF4
Sox4
mRNA level
Nanog

Supplementary Fig.3 Knock-down of USP16 promotes cell proliferation and induces anoikis resistance. Cell proliferation assays in Huh7 (**a**) and PLC/PRF/5 cells (**b**) with USP16 knock-down. (**c**) Cells were cultured on ultra-low adherent plates for three days and examined by FACS after Annexin V/7-AAD staining. (**d**)The images of TUNEL staining were shown (Bar, 100μm). Data represent mean±S.D, *p<0.05, **p<0.01, determined by *t*-test. (**e**) The mRNA levels of the indicated genes were analysed through real-time PCR in PLC/PRF/5 cells with or without USP16 knock-down, mean±S.D, *p<0.05, **p<0.01, ***p<0.001, determined by *t*-test.

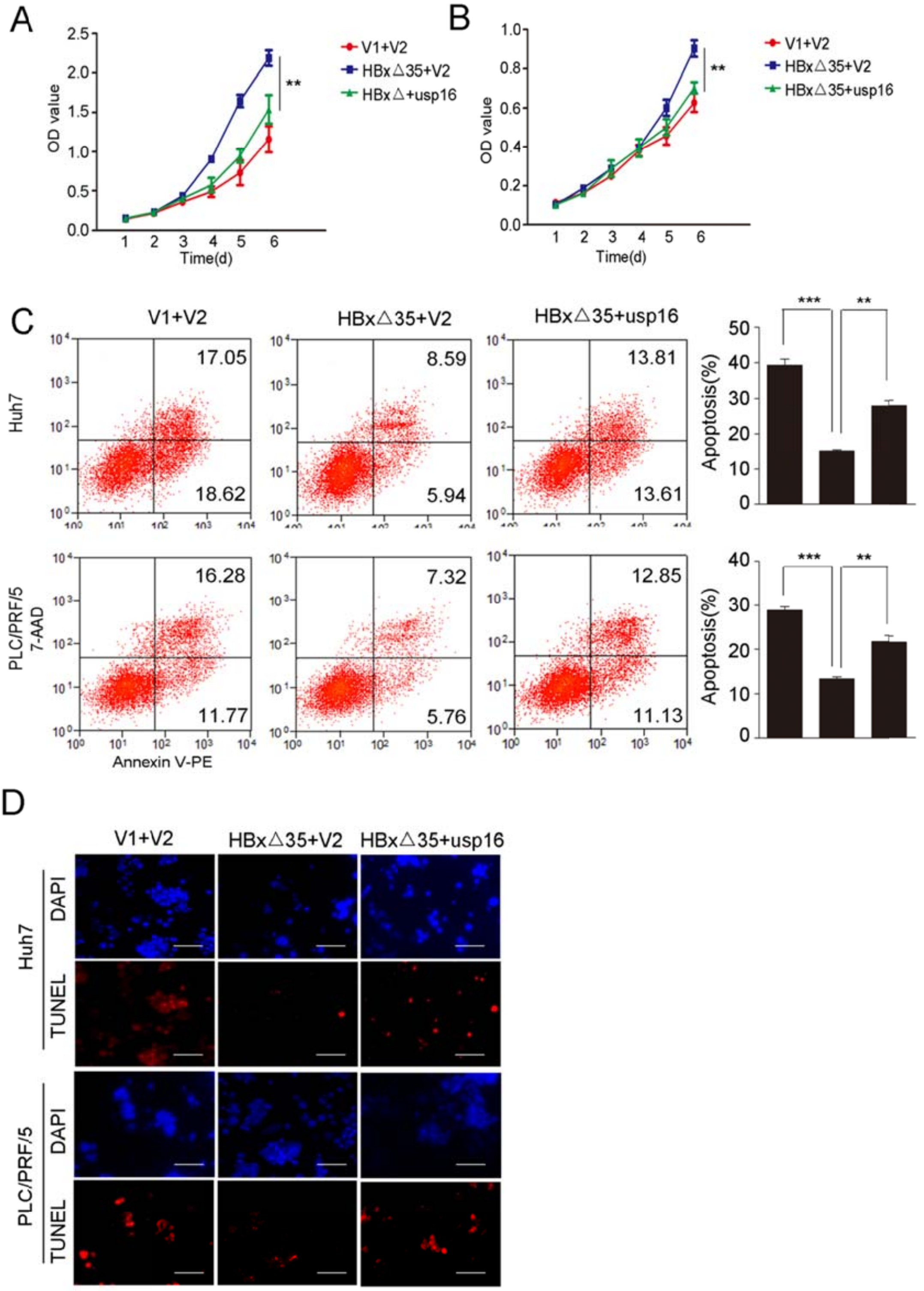
A
OD value
Time(d)
V1+V2
HBx△35+V2
HBx△+usp16
**
B
OD value
Time(d)
V1+V2
HBx△35+V2
HBx△35+usp16
**
C
V1+V2
HBx△35+V2
HBx△35+usp16
Huh7
17.05
18.62
8.59
5.94
13.81
13.61
Apoptosis(%)
***
**
PLC/PRF/5
7-AAD
16.28
11.77
7.32
5.76
12.85
11.13
Annexin V-PE
D
V1+V2
HBx△35+V2
HBx△35+usp16
Huh7
DAPI
TUNEL
PLC/PRF/5
DAPI
TUNEL

Supplementary Fig.4 Restored USP16 expression counteracts HBx△35-mediated promotion of cell proliferation and resistance to anoikis. (**a-b**) MTT assays were for the indicated Huh7 cells (**a**) and the indicated PLC/PRF/5 (**b**) cells. (**c**) Cells were cultured on ultra-low adherent plates for 3 days and examined by FACS after Annexin V/7-AAD staining. (**d**) The images of TUNEL staining of are shown (Bar, 100μm). Data represent mean±S.D, **p<0.01, ***p<0.001, determined by *t*-test.

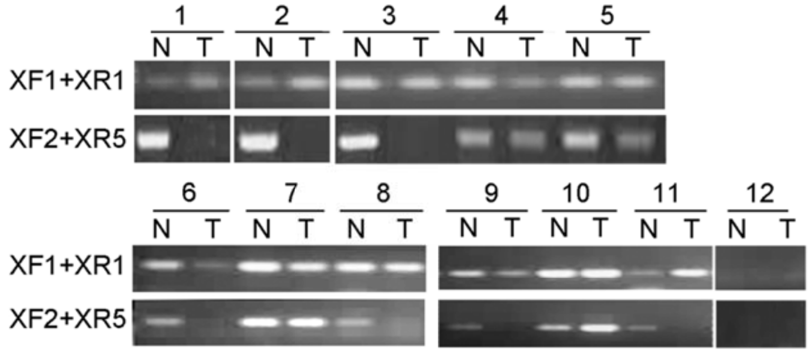

Supplementary Fig.5 Representative images of HBx status in HCC samples. HBx status in the paired HCCs and non-tumour tissues was investigated by RT-PCR. Primers used (XF1, XF2, XR1 and XR5) were listed in Table S1. PCR products were analysed by electrophoresis on a 1.5% agarose gel stained with EB. N: paired non-tumour tissue, T: tumour tissue.
